# Complete organellar genomes of six *Sargassum* species and development of species-specific markers

**DOI:** 10.1038/s41598-022-25443-4

**Published:** 2022-12-05

**Authors:** Yong Jin Lee, Yea Dam Kim, Yo Ram Uh, Yeon Mi Kim, Tae-Ho Seo, Sung-Je Choi, Cheol Seong Jang

**Affiliations:** 1 Plant Genomics Laboratory, Interdisciplinary Program in Smart Agriculture, Kangwon National University, Chuncheon, Republic of Korea; 2 Agriculture and Life Sciences Research Institute, Kangwon National University, Chuncheon, Republic of Korea; 3 Coastal Production Institute, Yeosu, Republic of Korea; 4 Korea National College of Agriculture and Fisheries, Jeonju, Republic of Korea

**Keywords:** DNA sequencing, Sequence annotation, Genetic markers

## Abstract

*Sargassum* is one of the most important brown algal genera that can be used as food and raw material for medicinal purpose, and has various beneficial effects. As the classification of *Sargassum* species is currently based on their morphological characteristics, organellar genome sequences of *Sargassum* would provide important information for accurate identification of species and developing species-specific markers. We sequenced the complete organellar genomes of six *Sargassum* species, including the first complete chloroplast genome sequences of *S. fulvellum*, *S. serratifolium*, *S. macrocarpum*, and *S. siliquastrum*, and the first complete mitochondrial genome sequences of *S. fulvellum*, *S. serratifolium*, and *S. macrocarpum*. The chloroplast genomes of the 6 *Sargassum* species contained 139 protein-coding genes (PCGs), and the mitochondrial genomes possessed 37 PCGs. A comparative study was performed between the newly sequenced organellar genomes and 44 other species belonging to class Phaeophyceae. Phylogenetic relationships using PCGs shared by Phaeophyceae species were constructed with IQ-TREE 2 using the maximum likelihood method. In addition, we developed real-time PCR markers based on SNPs to distinguish the 6 *Sargassum* species. Our results provide useful information for establishing phylogenetic relationships between brown algae.

## Introduction

*Sargassum* is a genus of brown algae (Phaeophyceae) that is distributed along the coastline of the lower intertidal zones, especially in northeast Asia. It forms marine ecosystems that provide food and habitat for diverse organisms in the sea^1^. Some species of *Sargassum*, such as *S. fusiforme* and *S. horneri* can be consumed and are raw materials for the development of medicine^2,3^, particularly in Asian countries. Recent studies have shown that, seaweed, including *Sargassum* have been known to have various beneficial compounds^4–6^; consequently, the seaweed market is constantly expanding, globally.

*Sargassum* species are distributed worldwide within intertidal and subtidal regions, forming dense submarine forests. Some species such as *S. horneri* can form harmful blooms known as “golden tides” and be spread on the ocean surface^7,8^.

The classification of brown algae, especially *Sargassum*, is based on the morphological characteristics of the thallus, stem, branches, and life-cycle types^9,10^. Unfortunately *Sargassum* species have limited morphological differences^7,11^ that can vary according to the environment, growth conditions, or developmental stages. Therefore, it is difficult to classify these species based on morphological characteristics alone.

Plastids and mitochondria are organelles that have evolved by endosymbiotic interactions with cyanobacteria and proteobacteria, respectively^12^. These organelles possess independent genomes; thus, the genetic information of these organelles is widely used as molecular markers to infer evolutionary relationships^13,14^. As next-generation sequencing (NGS) technologies have become cheaper and faster, a greater number of complete organellar genome sequences have been published. The *Sargassum* genus contains over 360 species^14^, but only a few organellar genomes of the genus have been completely sequenced despite the development of sequencing technologies.

In this study, we examined 6 *Sargassum* species (*S. confusum*, *S. fulvellum*, *S. horneri*, *S. macrocarpum*, *S. serratifolium*, and *S. siliquastrum*). These 6 species are used as raw materials for food, medicine, and therapeutic products in Korea and their economic value is growing^15^. Unfortunately, raw and processed materials of different brown algae species are typically mixed during the manufacturing process, due to the difficulties in morphological identification, which can lead to deterioration in product quality. Various Sargassum species have been taxonomically misclassified before (i.e., *S. macrocarpum* and *S. serratifolium* were thought to be the same species in Korea and Japan in the 1980s)^16^. In addition, many brown macroalgae within *Sargassum* are invasive, and there is a need for accurate identification to determine the risk. Since the classification of *Sargassum* species has recently improved with the use of NGS platforms for sequencing organellar genomes, the precise identification of the species is possible. This will prevent mixing and contamination by other cheap brown algae, thus improving consumers’ health and safeguarding their rights.

Here, we sequenced, assembled, and annotated the organellar genomes of 6 *Sargassum* species, including the first complete genome sequence of both the chloroplast and mitochondria of *S. fulvellum*, *S. macrocarpum*, and *S. serratifolium*, and the chloroplast of *S. siliquastrum* using the Illumina sequencing platform. Based on the genome structures and gene information of these organellar genomes, we conducted comparative studies and reconstructed their phylogenetic relationships with other Phaeophyceae species. Furthermore, we developed real-time PCR markers based on SNP that can distinguish each of the 6 species to prevent contamination of *Sargassum* in processed foods with other brown algae. As the demand of *Sargassum* for human consumption and as a raw material for medicine is growing^17,18^, it is important to distinguish each species using quick and easy methods. We demonstrated that sequence variations in the chloroplast and mitochondrial genomes of *Sargassum* species can distinguish between many target species. The obtained results provide valuable information for promoting the phylogenetics of brown algae and for developing molecular markers.

## Materials and methods

### Sargassum samples collection

Algal thalli were collected of *S. confusum* from Chujado island, Jeju, Republic of Korea (33°57′ N, 126°17′ E), *S. fulvellum* and *S. horneri* from Jeopdo island, Jeollanamdo, Republic of Korea (34°22′ N, 126°18′ E), *S. macrocarpum* from Geumodo island, Jeollanamdo, Republic of Korea (34°31′ N, 127°46′ E), *S. serratifolium* from Seongsan, Jeju, Republic of Korea (33°27′ N, 126°55′ E), and *S. siliquastrum* from Sikdo, Jeollabukdo, Republic of Korea (35°37′ N, 126°17′ E). All the species samples were collected and identified according to the morphological characteristics (Fig. S1) by professional experts (Sung-Je Choi & Tae-Ho Seo) in Korean algae. Permission to collect samples was granted by the Ministry of Oceans and Fisheries, Republic of Korea. Our studies were complied with local and national regulations and following Kangwon National University (Chuncheon, Republic of Korea) and the Ministry of Oceans and Fisheries (Sejong, Republic of Korea) regulations.

### DNA isolation and organellar genome sequencing, assembly, and annotation

The total *Sargassum* genomic DNA was extracted from the blades using the Exgene™ Plant SV Kit (GeneAll®, Seoul, Korea) according to the manufacturer’s instructions. Paired-end libraries of the samples were constructed and subsequently sequenced on the Illumina NovaSeq 6000 platform according to the manufacturer’s instructions. Raw reads were quality- and adapter-trimmed for GetOrganelle^19^ and only adapter-trimmed for NOVOPlasty^20^, for de novo assembly using Trimmomatic^21^. The organellar genomes were reconstructed and manually corrected using assembled sequences, by comparing the two assemblers. Annotation of the organellar genome was performed using GeSeq^22^ and Geneious Prime software^23^ with a custom reference GenBank database of 44 Phaeophyceae species (Table S1). The open reading frames of protein-coding genes (PCGs) were identified by manually comparing reference GenBank data and ‘Find ORFs’ function in Geneious software. tRNA genes were predicted using tRNAscan-SE^24^ v2.0.7 annotator in GeSeq with default parameters. Circular organellar genome maps of *Sargassum* were drawn using OGDRAW software^25^.

### Referential genome modification

We selected 44 Phaeophyceae species (two accessions for *Lessonia spicata* organellar genomes) (Table S1) for which chloroplast and mitochondrial genome data were available. All chloroplast and mitochondrial genomes were modified to start with the *ycf37* gene and *rnl* rRNA gene, respectively, for convenience in comparative analysis. Additionally, as some reference genomes possessed old gene names in their annotation, we provisionally re-annotated them using the 6 newly constructed *Sargassum* organellar genomes in this study to standardize gene names manually from old to new, (e.g., *ycf3* to *pafI*, *ycf40* to *thiS*) for comparative analysis and identification of shared genes among Phaeophyceae species.

### Repeat sequences analysis

Simple sequence repeats (SSRs) were detected using MISA^26^ with the parameters set at 10 > for mono-, > 5 for di- and tri-, > 3 for tetra-, penta-, and hexa-nucleotide SSRs. Dispersed repeats were identified using REPuter^27^ with parameters of Hamming distance 3, sequence identity ≥ 90%, and minimum repeat size ≥ 30 bp.

### Comparative analysis

The progressiveMauve^28^ alignment tool was used to analyze gene arrangement. The mVISTA software^29^ was used to analyze the divergences among the assembled *Sargassum* chloroplast and mitochondrial genomes, and the selected reference Phaeophyceae species in the Shuffle-LAGAN mode.

### Codon usage analysis

The codon usage bias was analyzed for all protein-coding genes (PCGs) using the CodonW program (http://codonw.sourceforge.net/), and the RSCU (relative synonymous codon usage) was visualized with the R script provided in Phylosuite^30^.

### Phylogenetic analysis

PCG sequences shared by 44 Phaeophyceae organellar genomes and six newly constructed *Sargassum* organellar genomes were used for phylogenetic analysis. Each of the 132 and 33 shared genes in the chloroplasts and mitochondria, respectively, were aligned using MAFFT^31^, and the ambiguously aligned regions in each alignment were deleted by trimAI^32^. The trimmed alignments of the chloroplast and mitochondrial genes were concatenated. We performed a maximum likelihood (ML) analysis using IQ-TREE 2^33^ under the GTR + F + I + G4 model, which was the best-fit model chosen according to Bayesian Information Criterion (BIC) by the implemented program ModelFinder in IQ-TREE 2, with 1,000 bootstrap and 1,000 replicates of the SH-aLRT test. The procedures from alignment to ML analysis were performed using the implemented and plugin functions of Phylosuite.

### Marker development for the identification of each of the 6 *Sargassum* species

The PCG sequences of the organellar genomes of the 6 *Sargassum* species were aligned using ClustalW. Primer pairs were designed with aligned PCG sequences based on SNPs using Beacon Designer™ (PRIMER Biosoft, Palo Alto, CA, USA). Quantitative real-time PCR for amplification was performed at 55–62 °C (depending on primer pairs) using the AccuPower® 2X GreenStar qPCR Master Mix (Bioneer, Daejeon, Korea) in a Quantstudio 3 Real-Time PCR System (Applied Biosystems, Foster City, CA, USA). The size of the species-specific amplified PCR products was confirmed using 1.5% agarose gel electrophoresis (Fig. 7).

## Results

### Characterization of organellar genomes of *Sargassum* species

**Figure 1:**
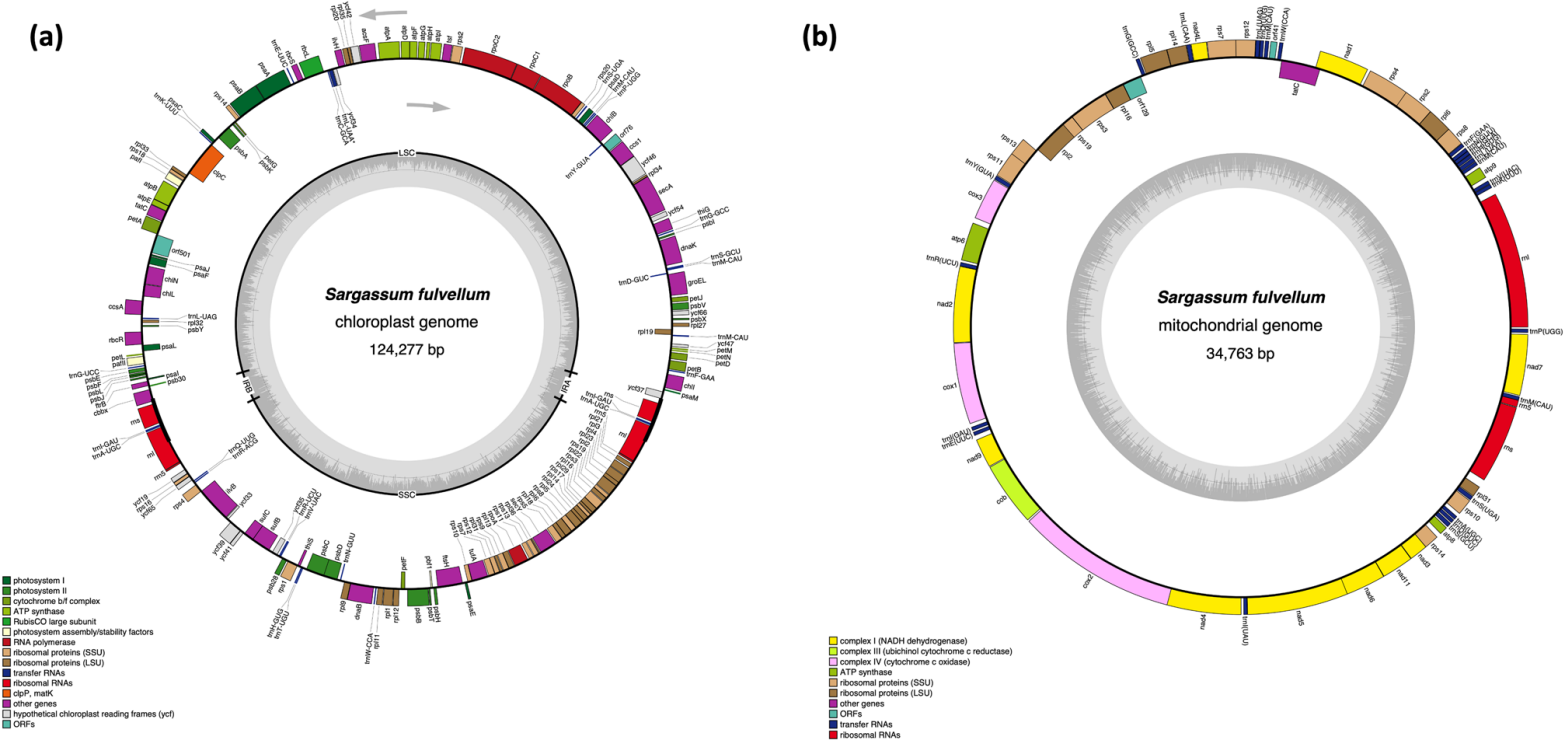
The organellar genome map of *Sargassum fulvellum*. The genes inside and outside of the circle are transcribed in clockwise and counterclockwise directions, respectively. The grey circle on the inside shows the GC content. The colored boxes represent gene functional groups. The thick lines in chloroplast genome map indicate the inverted repeats (IRa and IRb), which separate the genome into small (SSC) and large (LSC) single-copy regions. The chloroplast and mitochondrial genome maps of the other 5 species (*S. confusum*, *S. horneri*, *S. macrocarpum*, *S. serratifolium*, and *S. siliquastrum*) are shown in Fig. S2 and Fig. S3, respectively. Circular organellar genome maps were drawn using OGDRAW version 1.3.1 (https://chlorobox.mpimp-golm.mpg.de/OGDraw.html).

**Figure 2:**
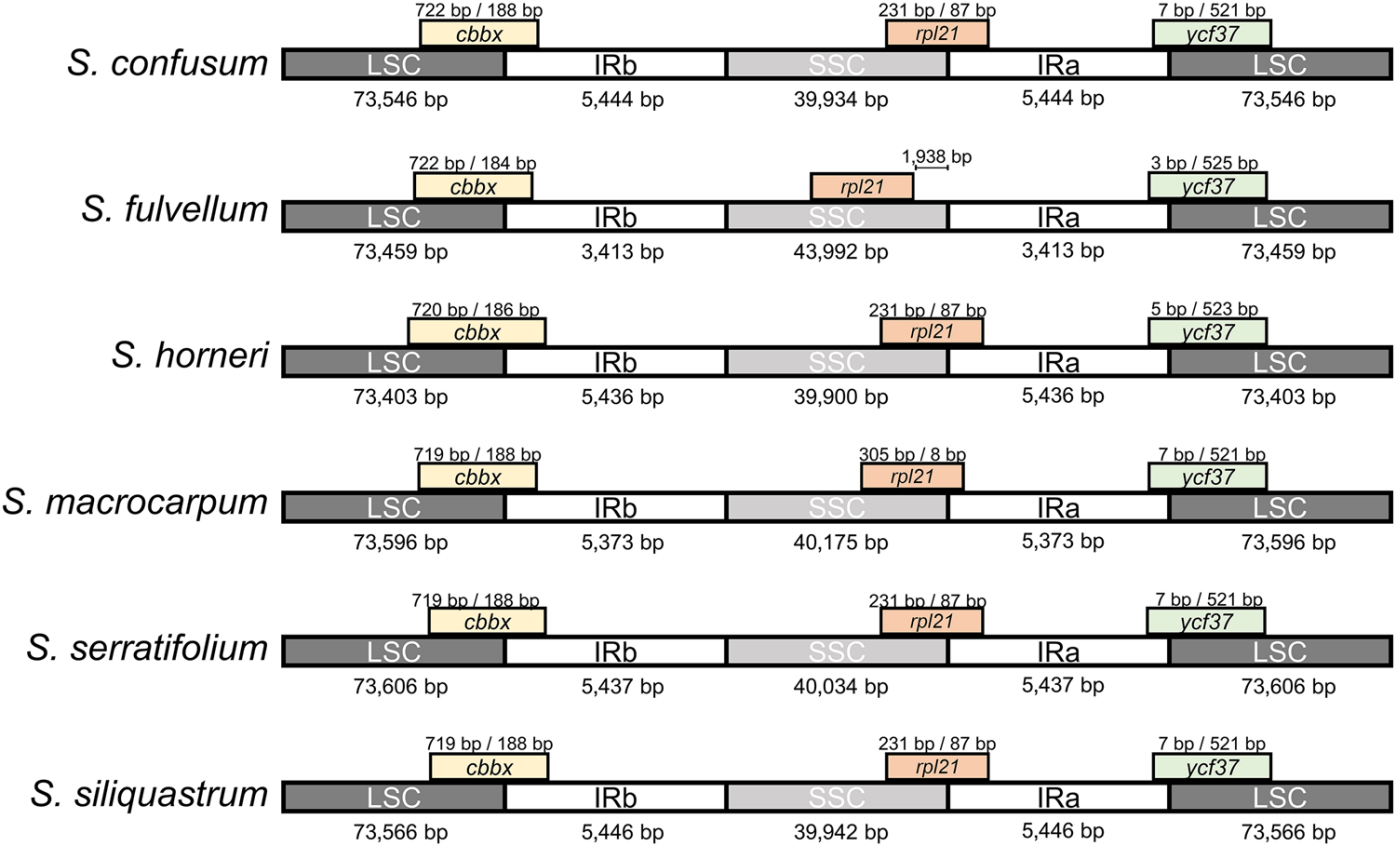
Comparison of the borders of large single copy (LSC), small single copy (SSC) and inverted repeat (IR) regions among the six *Sargassum* chloroplast genomes.

The DNA libraries were sequenced on Illumina platform, and we obtained average 25.3 Gb (from 24.2 Gb of *S. horneri* to 29.2 Gb of *S. macrocarpum*) of paired-end sequencing data, comprising from 67,195,726 (*S. serratifolium*) to 81,345,526 (*S. macrocarpum*) raw reads. Read coverage of each assembly varied from 466 to 1861X in chloroplast genomes and from 67 to 4540X in mitochondrial genomes. Among the 6 *Sargassum* species in this study, the organellar genomes of *S. fulvellum*, *S. serratifolium*, and *S. macrocarpum* were the first to be completely sequenced, assembled, and annotated organellar genomes in both chloroplast and mitochondria. Here we provided the organellar genome maps of *S. fulvellum* as Fig. 1 according to the alphabetical order of species name among the three species, and the organellar genome maps of the other 5 species were provided as Fig. S2 (chloroplast) and Fig. S3 (mitochondria). The chloroplast genomes of the 6 *Sargassum* species were assembled into a typical quadripartite circular structure containing large single copy (LSC) and small single copy (SSC) regions separated by 2 copies of an inverted repeat (IR) region, with sizes ranging from 124,175 to 124,517 bp (Fig. 1a, Fig. S2, and Table 1). The length of the LSC ranged from 73,403 bp (*S. horneri*) to 73,606 bp (*S. serratifolium*), while those of SSC and IRs were approximately 40,000 bp (39,900–40,175 bp) and 5400 bp (5373–5446 bp), respectively, with the exception of *S. fulvellum* (43,992 bp for SSC and 3413 bp for IRs). The GC content ranged from 30.3% to 30.6%. All 6 *Sargassum* species’ chloroplast genomes encoded 139 unique PCGs and 2 copies of each 3 rRNA genes. The chloroplast genomes encoded 28 tRNA genes (excluding *S. horneri* which had 29 tRNA genes), including 23 or 24 unique tRNA genes (Table 1). The IR regions and SC (single copy) region boundaries were analyzed, and high similarity was found among the six *Sargassum* species, with the exception of the SSC–IRa boundary of *S. fulvellum* (Fig. 2). The comparative location of the gene *rpl21* (overlapped on the SSC–IRa boundary of the other 5 species) was almost the same in all six *Sargassum* species. However, the SSC of *S. fulvellum* was approximately 4 kbp longer than that of other species, and consequently, the *rpl21* gene was located in the SSC region of *S. fulvellum*. Total chloroplast genes were classified according to their functions, including photosynthesis-related genes, genetic systems, RNA genes, genes with other functions, and those with unknown functions (Table S2).

**Table 1:** General features of complete organellar genomes of six *Sargassum* species.

Organelle	Characteristic	*S. confusum* (cp: ON660588, mt: ON675444)	*S. fulvellum* (cp: ON675439, mt: ON675445)	*S. horneri* (cp: ON675440, mt: ON675446)	*S. macrocarpum* (cp: ON675441, mt: ON675447)	*S. serratifolium* (cp: ON675442, mt: ON675448)	*S. siliquastrum* (cp: ON675443, mt: ON675449)
Chloroplast	Genome size (bp)	124,368	124,277	124,175	124,517	124,514	124,400
	GC content (%)	30.3	30.5	30.6	30.5	30.4	30.4
	LSC length (bp)	73,546	73,459	73,403	73,596	73,606	73,566
	SSC length (bp)	39,934	43,992	39,900	40,175	40,034	39,942
	IR length (bp)	5444	3,413	5,436	5,373	5,437	5,446
	Protein coding genes no. (unique)	139 (139)	139 (139)	139 (139)	139 (139)	139 (139)	139 (139)
	tRNA genes no. (unique)	28 (23)	28 (24)	29 (24)	28 (23)	28 (23)	28 (23)
	rRNA genes no. (unique)	6 (3)	6 (3)	6 (3)	6 (3)	6 (3)	6 (3)
Mitochondria	Genome size (bp)	34,719	34,763	34,620	34,766	34,793	34,721
	GC content (%)	36.6	36.6	36.2	36.6	36.6	36.6
	Protein coding genes no. (unique)	37 (37)	37 (37)	37 (37)	37 (37)	37 (37)	37 (37)
	tRNA genes no. (unique)	25 (24)	25 (23)	25 (23)	25 (23)	25 (23)	25 (23)
	rRNA genes no. (unique)	3 (3)	3 (3)	3 (3)	3 (3)	3 (3)	3 (3)

The lengths of the mitochondrial genomes ranged from 34,620 to 34,793 bp, with total GC contents ranging from 36.2% to 36.6% (Fig. 1b, Fig. S3, and Table 1). All mitochondrial genomes contained 37 unique PCGs and 3 rRNA genes. Twenty-five tRNA genes were encoded within all 6 *Sargassum* mitochondrial genomes, with 23 unique tRNA genes (except *S. confusum* with 24 unique tRNA genes) (Table 1). The mitochondrial genes involved in oxidative phosphorylation, genetic systems, RNA genes, genes with other functions, and genes whose functions are unknown are listed in Table S2.

### Repeat and simple sequence repeat (SSR) analysis

**Figure 3:**
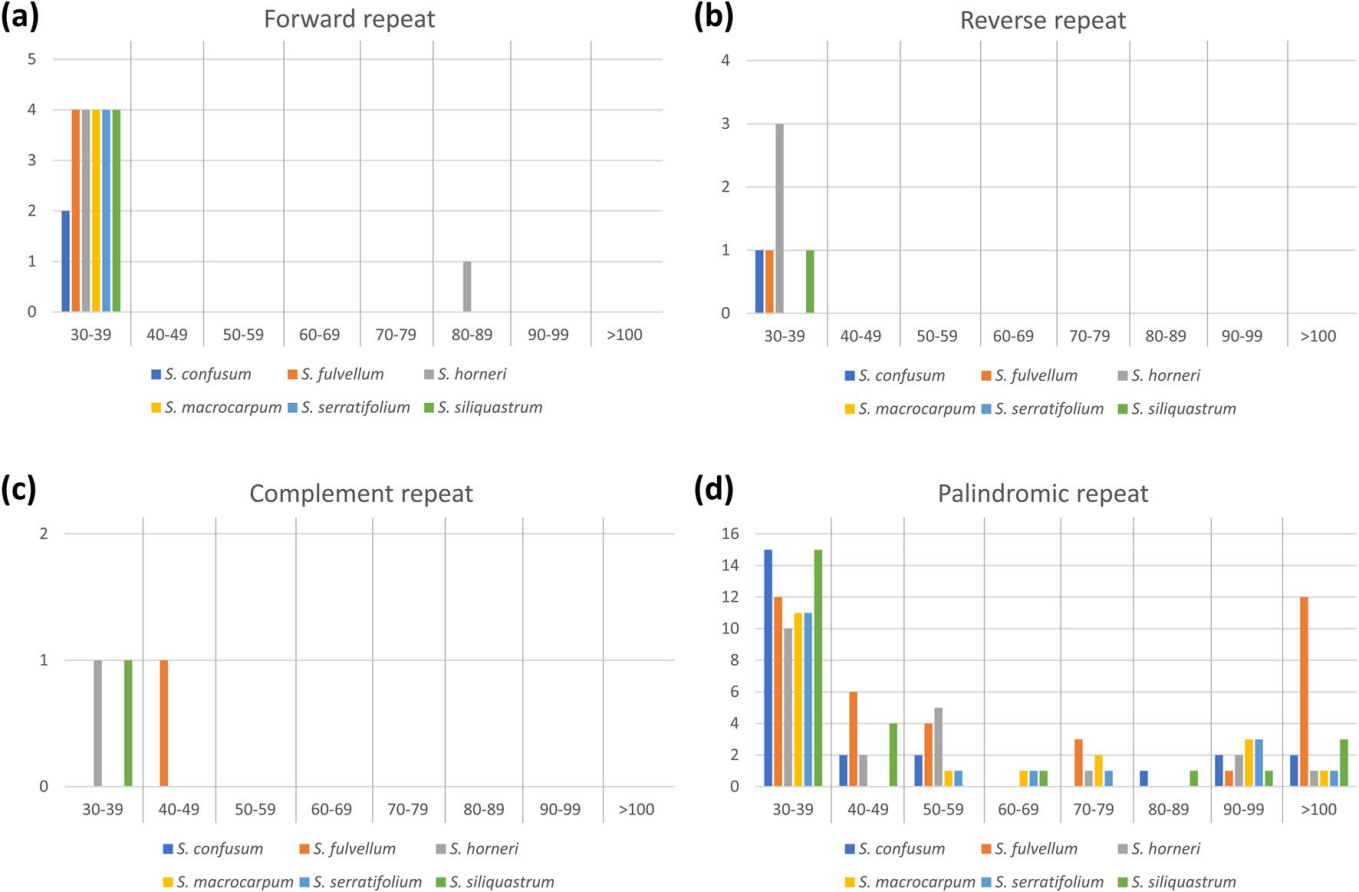
The longer repeat analysis of chloroplast genomes of the six *Sargassum* species. Frequency of (**a**) forward, (**b**) reverse, (**c**) complete, (**d**) palindromic repeats. The x-axis: repeat length; the y-axis: copy number.

In total, 39 (*S. confusum*), 49 (*S. fulvellum*), and 43 (*S. horneri*, *S. macrocarpum*, *S. serratifolium*, and *S. siliquastrum*) SSRs, respectively were detected in *Sargassum* chloroplast genomes. Among these, the most abundant SSRs were mononucleotide repeats (Table 2). In addition, A/T mono-nucleotide repeat units existed in all 6 *Sargassum* chloroplast genomes, but C/G repeats existed only in *S. macrocarpum* and *S. serratifolium*. The di-nucleotide repeat unit of AG/CT was only found in *S. fulvellum*, whereas the AT/AT repeat unit was found in more than 10 (10 to 14 repeats) repeats in all six species. The chloroplast genomes of the six species had under 10 repeats of tri-, tetra-, penta-, and hexa-nucleotide repeat units. Interestingly, the repeat units containing G or C occurred only once in all SSR types, with the exception of 2 repeats of AATG/ATTC tetra-nucleotide repeats in *S. fulvellum*. Dispersed long repeats of forward, reverse, complement, and palindrome were also detected in the chloroplast genomes (Fig. 3, the x-axis is the repeat length and the y-axis is the copy numbers). In *Sargassum* species, the most common repeat type was palindromic repeats, which accounted for 82% of the total repeats, followed by forward repeats (13%). Palindromic repeats with a range of 30–39 bp were the most abundant repeats. In particular, 12 copies of more than 100 bp palindromic repeat were abundant in *S. fulvellum* but only one to three copies were found in the other five species. Only six and three copies were found in the reverse and complement repeats, respectively over the six *Sargassum* chloroplast genomes.

**Table 2:** Types and numbers of SSRs in the organellar genomes of six *Sargassum* species.

Organelle	SSR type	Repeat unit	Species					
			*S. confusum*	*S. fulvellum*	*S. horneri*	*S. macrocarpum*	*S. serratifolium*	*S. siliquastrum*
Chloroplast	Mono	A/T	20	22	21	18	18	23
		C/G	0	0	0	1	1	0
	Di	AG/CT	0	1	0	0	0	0
		AT/AT	11	13	10	14	14	11
	Tri	AAT/ATT	1	2	1	1	1	1
		ATC/ATG	0	1	0	0	0	0
	Tetra	AAAT/ATTT	4	4	6	6	6	2
		AAAG/CTTT	0	1	1	0	0	1
		AATT/AATT	1	3	2	2	2	1
		AATG/ATTC	0	2	0	1	1	0
	Penta	AAAAT/ATTTT	0	0	1	0	0	1
		AATAT/ATATT	0	0	0	0	0	1
	Hexa	AAAGAT/ATCTTT	1	0	0	0	0	0
		AACTCC/AGTTGG	0	0	1	0	0	1
		AGATAT/ATATCT	1	0	0	0	0	1
Mitochondria	Mono	A/T	11	10	10	10	10	11
		C/G	0	0	0	0	0	1
	Di	AC/GT	0	1	0	1	1	0
		CG/CG	0	0	0	1	1	0
	Tetra	AAAC/GTTT	0	1	0	0	0	0
		AAAT/ATTT	1	1	1	1	1	1
		AGAT/ATCT	1	0	0	0	0	1
	Hexa	AAAGGG/CCCTTT	0	1	0	1	1	0

Mono-nucleotide SSRs with A/T repeats were found in *Sargassum* mitochondrial genomes (10–11 copies) and only one C/G repeat was found in *S. siliquastrum* (Table 2). There were no tri- and penta-nucleotide SSRs in any of the 6 *Sargassum* mitochondrial genomes. *S. confusum* and *S. siliquastrum* had one repeat of each unit of tetra- and hexa-nucleotide SSRs (AAAT/ATTT and AAAGGG/CCCTTT, respectively). *S. fulvellum* had one repeat of AC/GT, AAAC/GTTT, AAAT/ATTT, and AAAGGG/CCCTTT SSRs, and *S. horneri* had one repeat of tetra-nucleotide SSR (AAAT/ATTT). *S. macrocarpum* and *S. serratifolium* had one repeat of two SSR units of di-nucleotide (AC/GT and CG/CG), one tetra-nucleotide (AAAT/ATTT), and one hexa-nucleotide (AAAGGG/CCCTTT). In the mitochondrial genomes, REPuter detected only 1 forward repeat with a length of 34 bp in *S. serratifolium* (data not shown).

### Codon usage analysis

**Figure 4:**
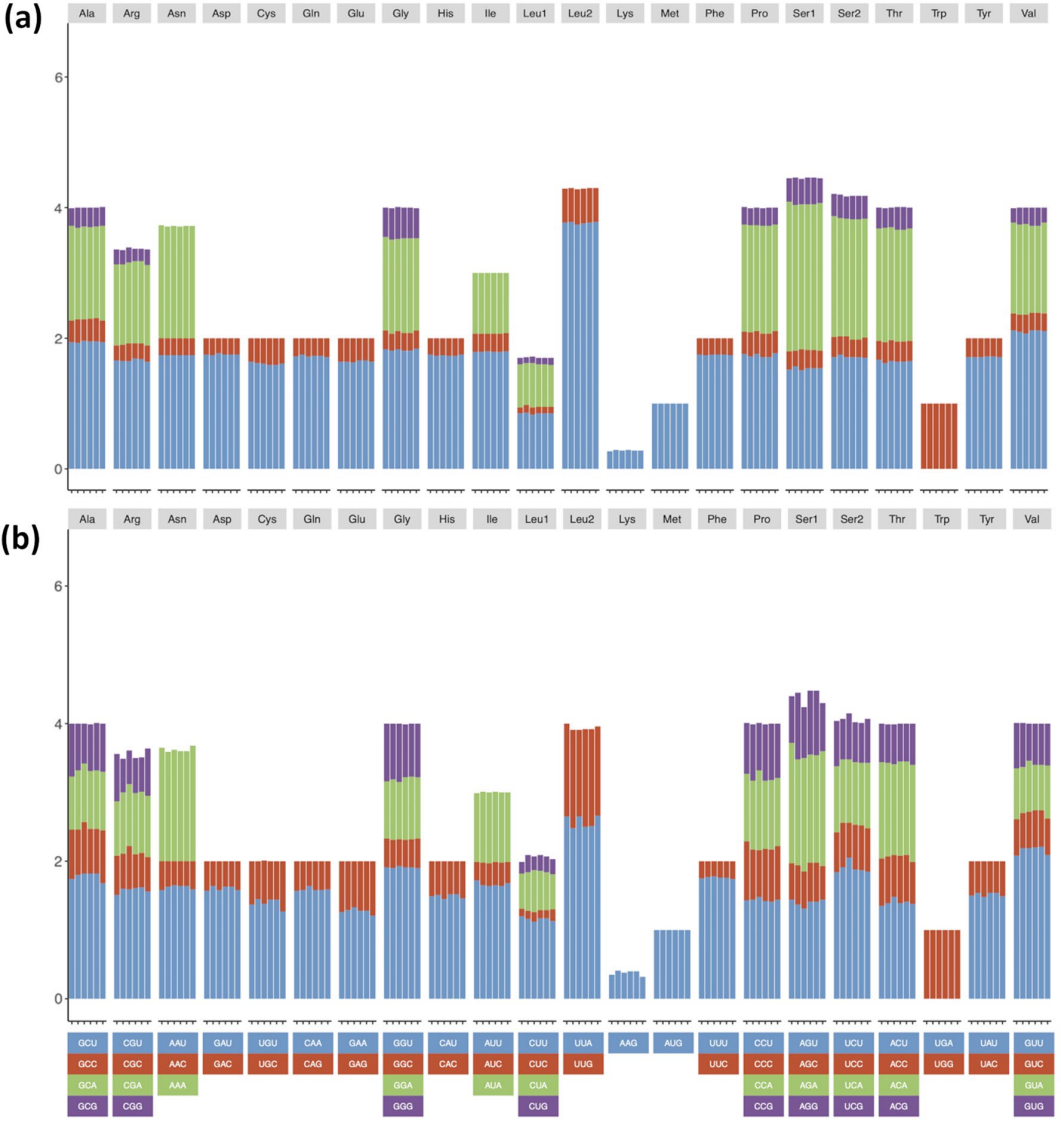
RSCU of organellar genomes of six *Sargassum* species. (**a**) Chloroplasts, (**b**) mitochondrial genomes.

In the *Sargassum* chloroplast genomes, PCGs consisted of 31,778 (*S. horneri*) to 31,814 (*S. confusum* and *S. siliquastrum*) codons. The total GC content (GC, 0.31 to 0.312) with GC at the first (GC1, 0.421 to 0.423), second (GC2, 0.345 to 0.346), and third (GC3, 0.164 to 0.168) codon position, respectively, were calculated (Supplementary Table S3). All codons with RSCU > 1 end with A and U in the chloroplast genomes of the six species (Fig. 4a). The results for the six *Sargassum* species showed that the chloroplast genomes tend to have A or U bases at the third codon position. G or C was biased toward a lower nucleotide frequency than A or U at the third codon position, indicating that the mutation towards A + U is a strong driving force for the chloroplast genome.

In the *Sargassum* mitochondrial genomes, the PCGs consisted of 8,983 (*S. horneri*) to 8,994 (*S. confusum* and *S. fulvellum*) codons in the mitochondria. The total GC content (GC, 0.353–0.359); GC content at the first (GC1, 0.414–0.421), second (GC2, 0.365–0.368), and third codon position (GC3, 0.279–0.289), respectively were calculated (Table S3). In the mitochondrial genomes, some codons with RSCU > 1 end with G (UUG in 6 *Sargassum* species) (Fig. 4b). However, all mitochondrial genomes tend to use A or U bases at the third codon position as chloroplast genomes.

### Comparative analysis of the *Sargassum* organellar genomes

The similarity percentage between organellar genome sequences of six *Sargassum* species was calculated using MAFFT. The sequence similarities were ranged from 93.36% (between *S. fulvellum* and *S. horneri*) to 99.95% (between *S. macrocarpum* and *S. serratifolium*) in chloroplast genomes, and from 87.91% (between *S. horneri* and *S. siliquastrum*) to 99.81% (between *S. macrocarpum* and *S. serratifolium*) in mitochondrial genomes (Table 3).

**Table 3:** Pairwise similarity (%) between organellar genome sequences of six *Sargassum* species.

Organelle	Species	*S. confusum*	*S. fulvellum*	*S. horneri*	*S. macrocarpum*	*S. serratifolium*
Chloroplast	*S. fulvellum*	94.26				
	*S. horneri*	95.36	93.36			
	*S. macrocarpum*	96.95	94.87	95.84		
	*S. serratifolium*	96.96	94.89	95.87	99.95	
	*S. siliquastrum*	99.14	94.25	95.32	96.87	96.89
Mitochondria	*S. fulvellum*	90.73				
	*S. horneri*	88.21	89.4			
	*S. macrocarpum*	90.84	99.5	89.5		
	*S. serratifolium*	90.79	99.4	89.43	99.81	
	*S. siliquastrum*	97.19	90.29	87.91	90.41	90.34

**Figure 5:**
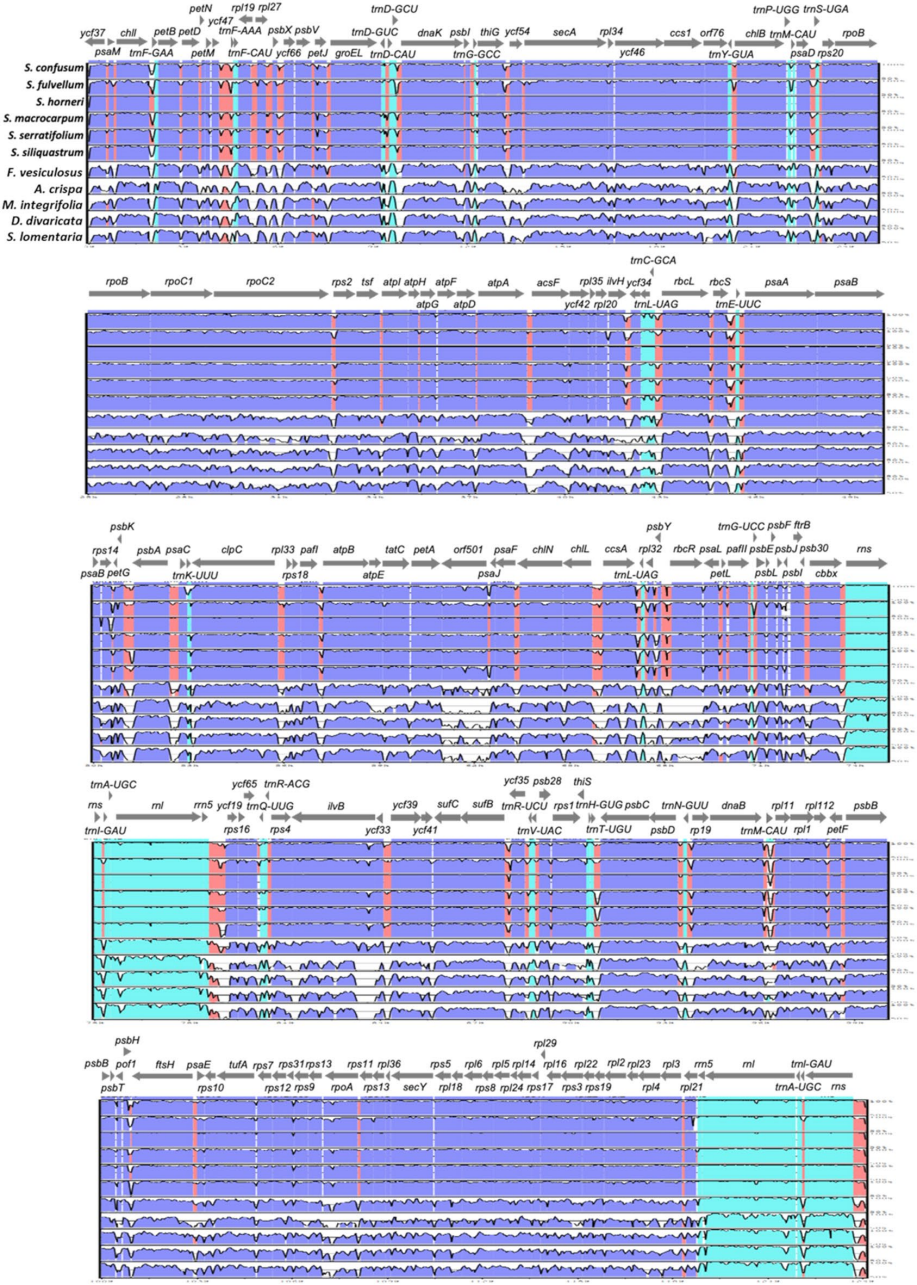
Alignment for sequence similarity among the 12 Phaeophyceae chloroplast genomes. Chloroplast genome of *S. horneri* (NC_02856) was used as the reference. Untranslated, conserved non-coding and coding regions were colored by skyblue, red, and blue, respectively.

We conducted a co-linear gene order analysis in the 44 Phaeophyceae reference chloroplast genomes deposited in the NCBI database (Fig. S4). The results showed that there were four gene order patterns in Phaeophyceae according to their taxonomic order, except *Chorda asiatica* with some inversion in the Laminariales gene order pattern. Furthermore, small variations in gene order were observed according to the taxonomic family. The analysis in mVISTA was conducted to identify the genome divergence of *Sargassum* species and six additional species from Phaeophyceae class, with *S. horneri* (NC_029856) as an alignment reference (Fig. 5). As illustrated in Fig. 5, the gene-coding regions (blue colored regions) and rRNAs (*rns*, *rrn5*, and *rnl* in skyblue colored regions) of all six *Sargassum* species had highly similar sequences, whereas tRNAs (the rest of skyblue colored regions) and spacer regions (red colored regions) showed large variations.

The mitochondrial genomes of Phaeophyceae class species showed fewer or no gene rearrangements than the chloroplast genomes (data not shown). However, the mVISTA results showed that the sequences of the Phaeophyceae class, including *Sargassum* species, were more varied than the sequences of chloroplasts (Fig. S5).

### Phylogenetic analysis

**Figure 6:**
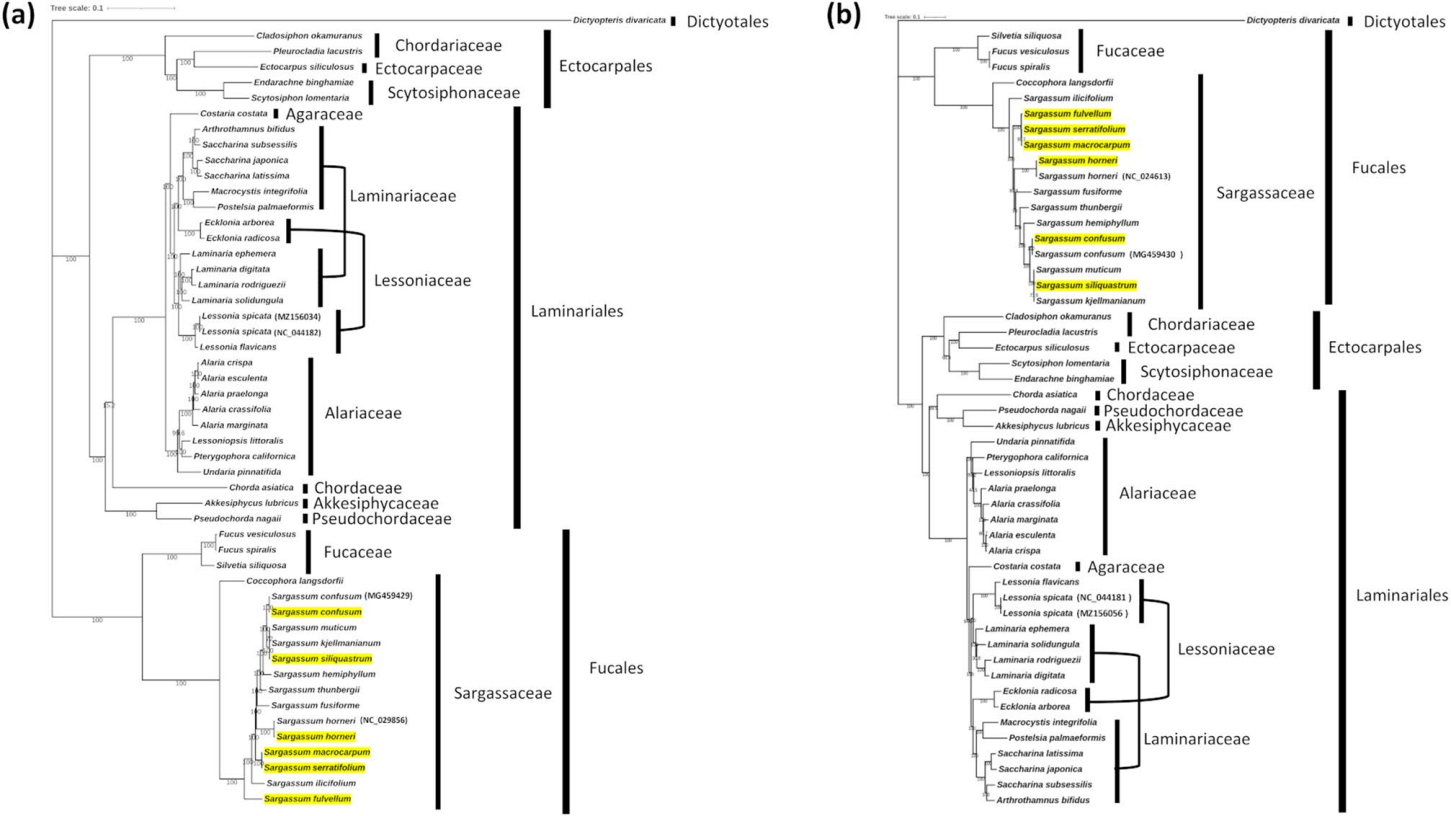
Phylogenetic analysis of 50 Phaeophyceae species including the 44 species deposited in NCBI and the six newly sequenced *Sargassum* genomes using Maximum Likelihood (ML) methods based on the concatenated shared PCG sequences of (**a**) chloroplast and (**b**) mitochondria. Yellow colored species are newly sequenced species. The numbers at internal nodes indicated maximum likelihood (ML) bootstrap values.

For the phylogenic analysis, 50 Phaeophyceae organellar genome sequences, including six *Sargassum* species, were used with *Dictyopteris divaricate* as an outgroup. The phylogenetic tree was constructed based on concatenated nucleotide sequences of 132 chloroplast PCGs (*orf501*, *petL*, *rbcR*, *rpl32*, *ycf34*, *ycf41*, and *ycf54* were excluded) and 33 mitochondrial PCGs (*atp8*, *orf39*, *orf129*, and *rps11* were excluded), shared by 44 Phaeophyceae species and 6 *Sargassum* species sequenced in this study. According to the phylogenetic analysis, all the species were classified as taxonomic orders matching the Ectocarpales, Fucales, Laminariales, and Dictyotales for outgroups in both trees based on chloroplasts and mitochondria (Fig. 6). However, some species belonging to the Laminariaceae and Lessoniaceae families showed mixed branch clusters. 6 species belonging to the Laminariaceae family (*A. bifidus*, *S. subsessilis*, *S. japonica*, *S. latissimi*, *M. integrifolia*, and *P. palmaeformis*) were more closely clustered with 2 species belonging to the Lessoniaceae family (*E. arborea* and *E. radicosa*) than with the other 4 *Laminaria* species (*L. ephemera*, *L. digitata*, *L. rodriguezii*, and *L. solidungula*) of the Laminariaceae family. The 4 *Laminaria* species were more closely clustered with the 2 *Lessonia* species (*L. spicata* and *L. flavicans*) of the Lessoniaceae family. Mixed clustering was found in phylogenetic trees constructed from both chloroplast and mitochondrial PCGs.

### Markers for identifying 6 *Sargassum* species

**Figure 7:**
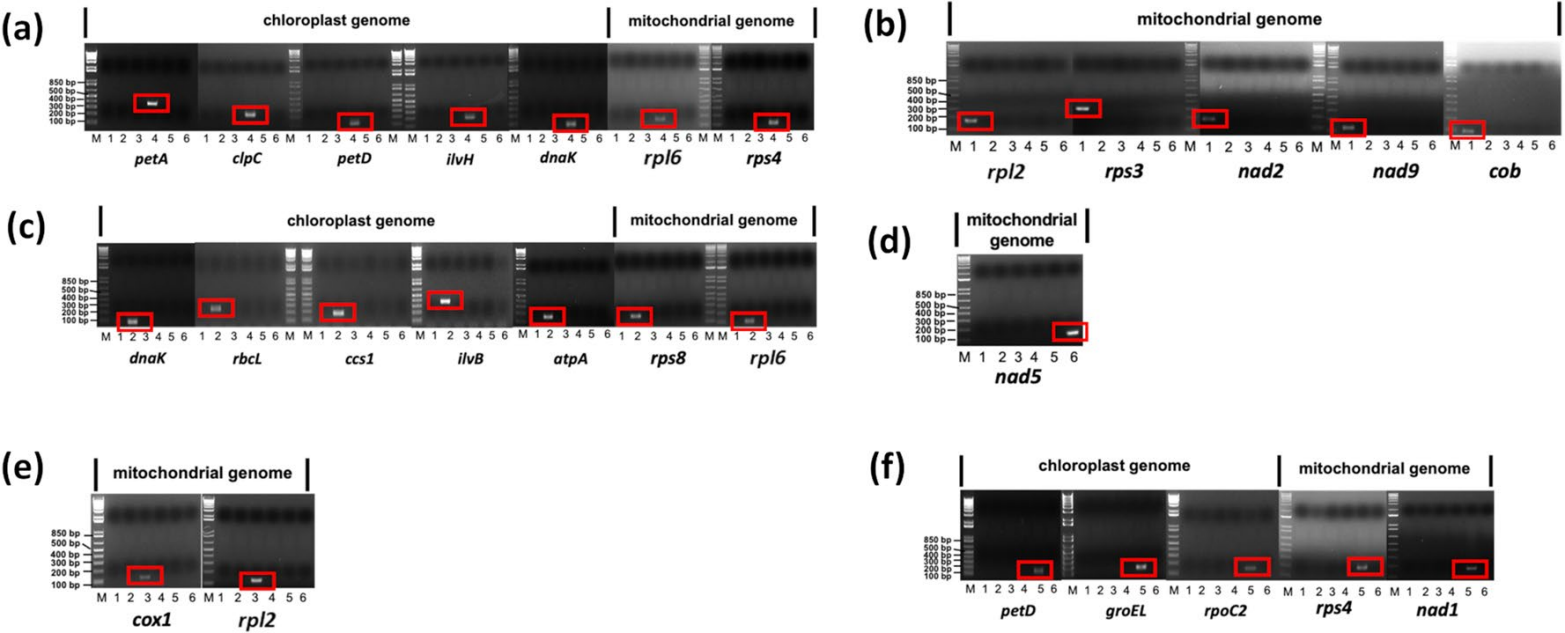
Development of quantitative real-time PCR based species-specific markers using SNPs of chloroplast and mitochondrial genomes. Target species are (**a**) *S. confusum*, (**b**) *S. fulvellum*, (**c**) *S. horneri*, (**d**) *S. macrocarpum*, (**e**) *S. serratifolium*, and (**f**) *S. siliquastrum*. Lane 1: *S. fulvellum*; 2: *S. horneri*; 3: *S. serratifolium*; 4: *S. confusum*; 5: *S. siliquastrum*; 6: *S. macrocarpum*, M: DNA ladder. Full-length gels are presented in Supplementary Fig. S6.

Although the SSRs and long repeats are dispersed in all organellar genomes of *Sargassum* species, the development of species-specific markers would be difficult because of their highly conserved border sequences and high AT content. Therefore, we focused on SNPs in PCG sequences for the development of species-specific markers. Based on the PCG sequence alignments, we developed 13 species-specific markers based on SNPs of chloroplast (five for *S. horneri* specific, five for *S. confusum* specific, and three for *S. siliquastrum* specific markers) and 14 markers based on SNPs of mitochondrial genomes (five for *S. fulvellum* and two each for *S. confusum*, *S. horneri*, *S. serratifolium*, and *S. siliquastrum*, and one for *S. macrocarpum*) (Fig. 7) with cut-off Ct ranging from 22 to 28 (Table S4).

## Discussion

Comparative analysis of the complete chloroplast genomes of the 6 *Sargassum* species showed high conservation in their structure, genome size, GC content, gene composition, and gene order, which is consistent with previously published data^34^. The chloroplast genomes of the 6 sequenced species had 139 unique PCGs and 3 unique rRNA genes. The notable difference among the 6 *Sargassum* chloroplast genomes was the size of inverted repeats in *S. fulvellum*, which was 3,413 bp (the other 5 species had inverted repeat regions of size ranging from 5,373 bp to 5,446 bp). The mitochondrial genomes of the *Sargassum* species studied shared 37 unique PCGs and three unique rRNA genes, and had identical gene orders. The mean sequence similarities were 96.05% in chloroplast genomes and 92.25% in mitochondrial genomes. The conservation among *Sargassum* genus is supported by the phylogenic analysis which showed that all members of *Sargassum* were clustered into one clade (Fucales) according to their taxonomic order. The consistent clustering of the clade suggests that there has been no rapid evolution of the *Sargassum* genus with respect to organellar genomes. Especially, *S. macrocarpum* and *S. serratifolium* showed extremely high similarity in sequences and were sister-species phylogenetically in both chloroplast and mitochondrial genomes.

Repeat sequences, including SSRs and long repeats, have been widely used in taxonomic analysis and phylogenetic relationships; and are also used as valuable markers in comparative genomics^35–37^. In this study, we identified several types of repeat sequences in the chloroplast genomes. The most abundant repeats were mono-nucleotide SSRs of A or T, followed by di-nucleotide SSRs. Unlike chloroplast genomes, limited repeats in the mitochondrial genomes were found, even though a number of mono-nucleotide SSRs in mitochondrial genomes were found, and only a few long repeats were detected. In addition, most repeat sequences contained extremely high AT content in both the chloroplast and mitochondrial genomes.

Codon usage is known to play an important role in gene expression levels and translation^38,39^. It provides valuable means for evolution by selection and mutation at the molecular level^40^. In the organellar genomes of the 6 *Sargassum* species, codon usage bias and RSCU based on the PCG sequences were calculated. In chloroplast genomes, AAA is the most common synonymous codon across species. Furthermore, UUA, GUU, and AGA in leucine, valine, and arginine, respectively, showed higher RSCU values (> 2.0), indicating that these synonymous codons were used more frequently than expected. The patterns of synonymous codon usage were more stable in *Sargassum* chloroplast genome, rather than in the mitochondrial genomes. Compared to other Phaeophyceae species, variations in synonymous usage patterns could be detected in both chloroplast and mitochondrial genomes. These specific patterns can be used to investigate the evolution of the brown algal family in future studies.

In this study, to further understand the *Sargassum* specific chloroplast genome characteristics, the complete chloroplast genomes of 44 Phaeophyceae species were aligned using the progressiveMauve program. The alignments revealed five patterns of gene rearrangements in the chloroplast genomes of Phaeophyceae species, according to taxonomic orders. Overall, comparative genomic analysis revealed that the *Sargassum* chloroplast genomes were relatively more conserved than those of the other species. In particular, the gene coding regions were highly conserved within the *Sargassum* species. The mitochondrial genomes of Phaeophyceae species used in the comparative study showed less variation in gene rearrangement than chloroplast genomes, whereas the sequence divergence in coding regions was relatively higher than that in the chloroplast genomes. Phylogenetic analysis also showed results similar to those of the comparative analysis, especially for gene rearrangements, except for *Chorda asiatica*, wherein the gene order patterns were clustered according to the taxonomic orders and even some families. However, our results showed that some species in the Laminariaceae and Lessoniaceae families are mixed in phylogenetic clustering. Previous reports have classified the species based on their morphological characteristics and phylogenetic analysis with a few marker genes^41^. In this study, we used 165 PCGs (132 chloroplast PCGs and 32 mitochondrial PCGs) shared in 50 species to reconstruct the phylogenetic tree, but we did not consider their morphological characteristics or other genetic factors. To verify their classifications, further studies should be conducted on the order Laminariales.

The results of the genome structures, codon usage analysis and comparative studies indicated that *Sargassum* species had highly conserved organelle genome sequences. Although, the repeat sequences showed some variation within the species, they showed high AT content, and these results were problematic for the development of species-specific molecular markers. Consequently, we focused on the SNPs in PCG sequences within the 6 *Sargassum* species, and developed 13 chloroplast and 14 mitochondrial genome based species-specific markers. As the genome sequence divergence results by mVISTA showed that mitochondrial genomes had more variations in their genome sequences, the development of mitochondrial markers was easier and more specific to the target species than the development of chloroplast markers. As the seaweed market is growing owing to its potential value, identifying precise target species has become a very important and sensitive issue, especially in Asia. The developed PCR markers are fast, easy, and specific for detecting target species. The *Sargassum* species are difficult to distinguish because of the similarities in both genetic and morphological characteristics. Additionally, many *Sargassum* species are the invasive species, and it makes the identification of accurate species more difficult. The species-specific patterns of genomic characteristics and molecular markers developed in this study can be used as valuable methods to define a target *Sargassum* species, and thus protecting consumers’ health and rights.

## Supplementary Information

Supplementary Information.

## Data Availability

The complete organelle sequences generated and analyzed during the current study are uploaded in NCBI GenBank (ON660588 and ON675439–ON675449).
