# Supplementary material for: Complete organellar genomes of six Sargassum species and development of species-specific markers
Source: Sci Rep. 2022 Dec 5;12:20981. doi: 10.1038/s41598-022-25443-4 (PMC9722929; doi:10.1038/s41598-022-25443-4)
Supplement: Supplementary file 1 — Supplementary Information. [file 41598_2022_25443_MOESM1_ESM.pdf]

Complete organelle genomes of six *Sargassum* species and development of species-specific markers

Yong Jin Lee[1,2], Yea Dam Kim[1], Yo Ram Uh[1], Yeon Mi Kim[1], Tae-Ho Seo[3], Sung-Je Choi[4] & Cheol Seong Jang[1,2]*

[1]Plant Genomics Laboratory, Interdisciplinary Program in Smart Agriculture, Kangwon National University, Chuncheon, Republic of Korea

[2]Agriculture and life Sciences Research Institute, Kangwon National University, Chuncheon, Republic of Korea

[3]Coastal Production Institute, Yeosu, Republic of Korea

[4]Korea National College of Agriculture and Fisheries, Jeonju, Republic of Korea

*Corresponding author,

E-mail: csjang@kangwon.ac.kr

Supplementary Material

Table S1. List of 44 species and accessions of chloroplast and mitochondrial genome used for comparative and phylogenetic analysis.
Table S2. Functional classification of genes encoded in six *Sargassum* organelle genomes.
Table S3. GC contents and codon compositions of six *Sargassum* organelle codons.
Table S4. Molecular markers based on quantitative PCR using SNPs in organelle genomes to distinguish target species.

Figure S1.
Figure S2.
Figure S3.
Figure S4.
Figure S5.
Figure S6.

Table S1. List of 44 species and accessions of chloroplast and mitochondrial genome used for comparative and phylogenetic analysis.

**Organism**	**Taxonomy (Class)**	**Order**	**Family**	**Genus**	**Chloroplast Accession**	**Mitochondria Accession**
Akkesiphycus lubricus	Phaeophyceae	Laminariales	Akkesiphycaceae	Akkesiphycus	MZ156027.1	MZ156045.1
Alaria crassifolia	Phaeophyceae	Laminariales	Alariaceae	Alaria	NC_058771.1	NC_058766.1
Alaria crispa	Phaeophyceae	Laminariales	Alariaceae	Alaria	NC_058768.1	NC_058763.1
Alaria esculenta	Phaeophyceae	Laminariales	Alariaceae	Alaria	NC_058770.1	NC_058765.1
Alaria marginata	Phaeophyceae	Laminariales	Alariaceae	Alaria	NC_058769.1	NC_058764.1
Alaria praelonga	Phaeophyceae	Laminariales	Alariaceae	Alaria	NC_058772.1	NC_058767.1
Arthrothamnus bifidus	Phaeophyceae	Laminariales	Laminariaceae	Arthrothamnus	MZ156043.1	MZ156049.1
Chorda asiatica	Phaeophyceae	Laminariales	Chordaceae	Chorda	MZ156037.1	MZ156050.1
Cladosiphon okamuranus	Phaeophyceae	Ectocarpales	Chordariaceae	Cladosiphon	NC_046005.1	NC_040224.1
Coccophora langsdorfii	Phaeophyceae	Fucales	Sargassaceae	Coccophora	NC_032288.1	NC_032287.1
Costaria costata	Phaeophyceae	Laminariales	Agaraceae	Costaria	NC_028502.1	NC_023506.1
Dictyopteris divaricata	Phaeophyceae	Dictyotales	Dictyotaceae	Dictyopteris	NC_036804.1	NC_043845.1
Ecklonia arborea	Phaeophyceae	Laminariales	Lessoniaceae	Ecklonia	MZ156038.1	MZ156048.1
Ecklonia radicosa	Phaeophyceae	Laminariales	Lessoniaceae	Ecklonia	MZ156040.1	MZ156054.1
Ectocarpus siliculosus	Phaeophyceae	Ectocarpales	Ectocarpaceae	Ectocarpus	NC_013498.1	NC_030223.1
Endarachne binghamiae	Phaeophyceae	Ectocarpales	Scytosiphonaceae	Endarachne	NC_038231.1	MG488291.1
Fucus spiralis	Phaeophyceae	Fucales	Fucaceae	Fucus	MG922855.1	MG922856.1
Fucus vesiculosus	Phaeophyceae	Fucales	Fucaceae	Fucus	FM957154.1	NC_007683.1
Laminaria digitata	Phaeophyceae	Laminariales	Laminariaceae	Laminaria	NC_044689.1	NC_004024.1
Laminaria ephemera	Phaeophyceae	Laminariales	Laminariaceae	Laminaria	MZ156035.1	MZ156055.1
Laminaria rodriguezii	Phaeophyceae	Laminariales	Laminariaceae	Laminaria	NC_057231.1	NC_057230.1
Laminaria solidungula	Phaeophyceae	Laminariales	Laminariaceae	Laminaria	NC_044690.1	NC_056140.1

Lessonia flavicans	Phaeophyceae	Laminariales	Lessoniaceae	Lessonia	NC_056288.1	NC_056287.1
Lessonia spicata	Phaeophyceae	Laminariales	Lessoniaceae	Lessonia	MZ156034.1	MZ156056.1
Lessonia spicata	Phaeophyceae	Laminariales	Lessoniaceae	Lessonia	NC_044182.1	NC_044181.1
Lessoniopsis littoralis	Phaeophyceae	Laminariales	Alariaceae	Lessoniopsis	MZ156033.1	MZ156066.1
Macrocystis integrifolia	Phaeophyceae	Laminariales	Laminariaceae	Macrocystis	NC_058274.1	NC_042669.1
Pleurocladia lacustris	Phaeophyceae	Ectocarpales	Chordariaceae	Pleurocladia	NC_032045.1	NC_032046.1
Postelsia palmaeformis	Phaeophyceae	Laminariales	Laminariaceae	Postelsia	MZ156031.1	MZ156060.1
Pseudochorda nagaii	Phaeophyceae	Laminariales	Pseudochordaceae	Pseudochorda	MZ156030.1	MZ156063.1
Pterygophora californica	Phaeophyceae	Laminariales	Alariaceae	Pterygophora	MZ156029.1	MZ156061.1
Saccharina japonica	Phaeophyceae	Laminariales	Laminariaceae	Saccharina	NC_018523.1	NC_013476.1
Saccharina latissima	Phaeophyceae	Laminariales	Laminariaceae	Saccharina	NC_049039.1	NC_026108.1
Saccharina subsessilis	Phaeophyceae	Laminariales	Laminariaceae	Saccharina	MZ156036.1	MZ156062.1
Sargassum confusum	Phaeophyceae	Fucales	Sargassaceae	Sargassum	MG459429.1	MG459430.1
Sargassum fusiforme	Phaeophyceae	Fucales	Sargassaceae	Sargassum	NC_048511.1	NC_024655.1
Sargassum hemiphyllum	Phaeophyceae	Fucales	Sargassaceae	Sargassum	MT873582.1	MT845205.1
Sargassum horneri	Phaeophyceae	Fucales	Sargassaceae	Sargassum	NC_029856.1	NC_024613.1
Sargassum ilicifolium	Phaeophyceae	Fucales	Sargassaceae	Sargassum	MW767830.1	KT272403.1
Sargassum kjellmanianum	Phaeophyceae	Fucales	Sargassaceae	Sargassum	OK323194.1	OK235333.1
Sargassum muticum	Phaeophyceae	Fucales	Sargassaceae	Sargassum	MW784166.1	NC_024614.1
Sargassum thunbergii	Phaeophyceae	Fucales	Sargassaceae	Sargassum	NC_029134.1	NC_026700.1
Scytosiphon lomentaria	Phaeophyceae	Ectocarpales	Scytosiphonaceae	Scytosiphon	NC_057081.1	NC_025240.1
Silvetia siliquosa	Phaeophyceae	Fucales	Fucaceae	Silvetia	NC_061768.1	MW485976.1
Undaria pinnatifida	Phaeophyceae	Laminariales	Alariaceae	Undaria	NC_028503.1	NC_023354.1

Table S2. Functional classification of genes encoded in six *Sargassum* organelle genomes.

Organelle	Category	Group of genes	Name of genes
Chloroplast	Photosynthesis related genes	Photosystem I	*psaA, psaB, psaC, psaD, psaE, psaF, psaI, psaJ, psaL, psaM*
		Assembly/stability of photosystem I	*pafI (ycf3), pafII (ycf4)*
		Photosystem II	*psbA, psbB, psbC, psbD, psbE, psbF, psbH, psbI, psbJ, psbK, psbL, psbT, psbV, psbX, psbY, psb28 (psbW), psb30 (ycf12)*
		Assembly/stability of photosystem II	*pbf1 (psbN)*
		Cytochrome c synthesis	*ccs1, ccsA*
		Cytochrome b/f complex	*petA, petB, petD, petG, petL, petM, petN*
		Electron transport	*petF, petJ*
		Carbon assimilation	*rbcL, rbcS, ilvB, ilvH, thiG, thiS (ycf40)*
		ATP synthase	*atpA, atpB, atpD, atpE, atpF, atpG, atpH, atpI*
		Light harvesting, chl biosynthesis, and redox system	*acsF, chlB, chlI, chlL, chlN, bas1 (ycf42), ftrB*
	Genetic system	RNA polymerase	*rpoA, rpoB, rpoC1, rpoC2*
		Ribosomal protein small subunit	*rps1, rps2, rps3, rps4, rps5, rps7, rps8, rps9, rps10, rps11, rps12, rps13, rps14, rps16, rps17, rps18, rps19, rps20*
		Ribosomal protein large subunit	*rpl1, rpl2, rpl3, rpl4, rpl5, rpl6, rpl9, rpl11, rpl12, rpl13, rpl14, rpl16, rpl18, rpl19, rpl20, rpl21, rpl22, rpl23, rpl24, rpl27, rpl29, rpl31, rpl32, rpl33, rpl34, rpl35, rpl36*
		Protein quality control	*clpC, dnaK, ftsH, groEL*
		Transcription factor	*rbcR*
		Maintenance	*dnaB*
		Translation	*tsf, tufA*

	Other	RubisCo expression protein	*cbbX*
		Protein transport	*secA, secY, tatC, sufB, sufC*
	RNA genes	Transfer RNAs	*trnA-UGC, trnC-GCA, trnD-GUC, trnE-UUC, trnF-AAA, trnF-GAA, trnG-GCC, trnG-UCC, trnH-GUG, trnI-GAU, trnK-UUU, trnL-UAA, trnL-UGA, trnM-CAU, trnN-GUU, trnP-UGG, trnQ-UUG, trnR-ACG, trnR-UCU, trnS-GCU, trnS-UGA, trnT-UGU, trnV-UAC, trnW-CCA, trnY-GUA*
		Ribosomal RNAs	*rnl x 2, rns x 2, rrn5 x 2*
	Unknown		*orf76, orf501, ycf19, ycf33, ycf34, ycf35, ycf37, ycf39, ycf41, ycf46, ycf47, ycf54, ycf65, ycf66*
Mitochondria	Oxidative phosphorylation	ATP synthase	*atp6, atp8, atp9*
		Cytochrome b	*cob*
		Cytochrome oxidase	*cox1, cox2, cox3*
		NAD dehydrogenase	*nad1, nad2, nad3, nad4, nad4L, nad5, nad6, nad7, nad9, nad11*
	Genetic system	Ribosomal protein small subunit	*rps2, rps3, rps4, rps7, rps8, rps10, rps11, rps12, rps13, rps14, rps19*
		Ribosomal protein large subunit	*rpl2, rpl5, rpl6, rpl14, rpl16, rpl31*
	RNA genes	Transfer RNAs	*trnA-UGC, trnC-GCA, trnD-GUC, trnE-UUC, trnF-GAA, trnG-GCC, trnH-GUG, trnI-UAU, trnK-UUU, trnL-CAA, trnL-UAA, trnL-UAG, trnM-CAU, trnN-GUU, trnP-UGG, trnQ-UUG, trnR-UCU, trnS-GCU, trnS-UGA, trnV-UAC, trnW-CCA, trnY-GUA*
		Ribosomal RNAs	*rnl, rns, rrn5*
	Other	Protein transport	*tatC*
	Unknown		*orf39, orf129*

Table S3. GC contents and codon compositions of six *Sargassum* organelle codons.

Organelle	Species	GC	GC1	GC2	GC3
Chloroplast	*S. confusum*	0.31	0.421	0.345	0.164
	S. fulvellum	0.312	0.423	0.345	0.167
	S. horneri	0.312	0.423	0.346	0.168
	S. macrocarpum	0.311	0.421	0.345	0.167
	S. serratifolium	0.311	0.421	0.345	0.166
	S. siliquastrum	0.311	0.421	0.345	0.166
Mitochondria	*S. confusum*	0.357	0.416	0.366	0.289
	S. fulvellum	0.359	0.421	0.368	0.287
	S. horneri	0.353	0.414	0.365	0.279
	S. macrocarpum	0.358	0.42	0.368	0.286
	S. serratifolium	0.358	0.42	0.368	0.286
	S. siliquastrum	0.357	0.417	0.366	0.289

Table S4. Molecular markers based on quantitative PCR using SNPs in organelle genomes to distinguish target species.

Organelle	Species	Target gene	Primer	Length (bp)	Sequence (5' → 3')	Fragment length (bp)	cut-off Ct	Tm (℃)
Chloroplast	*S. confusum*	clpC	clpC_F	18	GTATTCTCTGACAGCATT	210	22	55
			clpC_R	20	GGTATATCAACAAATCACAT			
		petA	petA_F	18	AAGTGGCTTAAATGTTGG	355	22	58
			petA_R	18	AGCTTGGATTTCTGTGAT			
		petD	petD_F	20	ATGGGACATAATTACTACGG	108	27	59
			petD_R	21	CATAACAGCTAAGCCAATAAC			
		dnaK	dnaK_2F	21	AGATTAGAGGGAATACCACTA	87	27	59
			dnaK_2R	21	TACTGATAATATCCCACTTGC			
	S. horneri	dnaK	dnaK_2F	20	ACAAATTCAGTAGTTGCCAT	79	27	59
			dnaK_2R	19	ATGGTGTTGTTCTAAGACC			
		ccs1	ccs1_F	22	CGTAGCAAAGTATTTACTACGC	220	22	62
			ccs1_R	20	CCCCAATGTGCTTCCCAGTA			
		ilvB	ilvB_F	19	ACTTATGGAGCAGAGAATG	380	22	59
			ilvB_R	23	GCTGATTTAAGTCTCGTGGAGCG			
		rbcL	rbcL_F	19	TGTATGAACGTGCAGAGTA	239	22	61
			rbcL_R	21	AGTACCAGCATGAATATGGTC			
		atpA	atpA_F	18	TACATCGGGTAATGACAC	148	22	57
			atpA_R	21	ACCAAATAATCCAATTTTACC			
	S. siliquastrum	petD	petD_F	16	TAGGGACTTTCGTGAT	97	26	55
			petD_R	20	GGTAAAATTTCTAAAGGTGT			
		groEL	groEL_F	17	GCGATTTTAACACAAGG	162	26	55

			groEL_R	18	ATCACACCGAGTTTTAAT			
		rpoC2	rpoC_3F	18	CAACGAGTAATGGGTTTA	136	26	55
			rpoC_3R	22	GTTTTAAAATTATCTCATGGTT			
Mitochondria	*S. confusum*	rp16	rp16_F	16	TTTAGGCGTCGTCGAT	154	28	60
			rp16_R	20	TTTAAGAACCAATTCCACGC			
		rps4	rps4_F	21	AAAAACTTTTTTGCGTCCAAA	113	28	60
			rps4_R	15	TGTGTCTACGGGTGG			
	S. fulvellum	rp12	rp12_F	24	GCATTATTTAATCCACAAAAGATT	166	24	58
			rp12_R	19	CACAAGGGATTGTTATAGG			
		rps3	rps3_F	20	CCAGTAAATGTCTGAATGAG	296	24	58
			rps3_R	17	TATCAAGGTTACACGCA			
		nad2	nad2_F	20	TTGGGAAGACTTTTTTTGTT	170	24	58
			nad2_R	19	AAACACTTTGAACTCCTTC			
		nad9	nad9_F	20	CGGATTTACGTCGAATTATT	126	24	58
			nad9_R	16	TAGGCTCACAGACAAC			
		cob	cob_F	16	GTCCGAATAAACGCTG	101	24	58
			cob_R	20	TTATGAGATATCTCTGGAGG			
	S. horneri	rps8	rps8_F	22	TAAGTTACGTAATAGCCTTTCT	169	28	59
			rps8_R	19	GATAGGAGATCCATCGGAA			
		rp16	rp16_F	17	GGTGTACTCGTGAGGTT	98	28	62
			rp16_R	21	AAACGGTATGCTCGTATACTT			
	S. macrocarpum	nad5	nad5_F	14	CAAGCCCGCCTATG	111	22	59
			nad5_R	22	ATTCGTAAAGAAGGGAAACTAA			
	S. serratifolium	cox1	cox1_F	19	CAATTCTTTCCTCAGTTGC	117	28	59

			cox1_R	17	CTGGAAAAGCAACATCC			
		rp12	rp12_F	20	TATTCCAATCCGATAACGAT	117	25	58
			rp12_R	19	ACGCAATAATAAAGGTTGG			
	S. siliquastrum	rps4	rps4_F	17	AGGCGACCTTTAGCTAA	180	28	61
			rps4_R	20	CCCATAAAACCGTCTTAGAC			
		nad1	nad1_F	16	GACTTCTTCAACCCTT	156	28	55
			nad1_R	19	CATTACCATAACTAAAGGG			

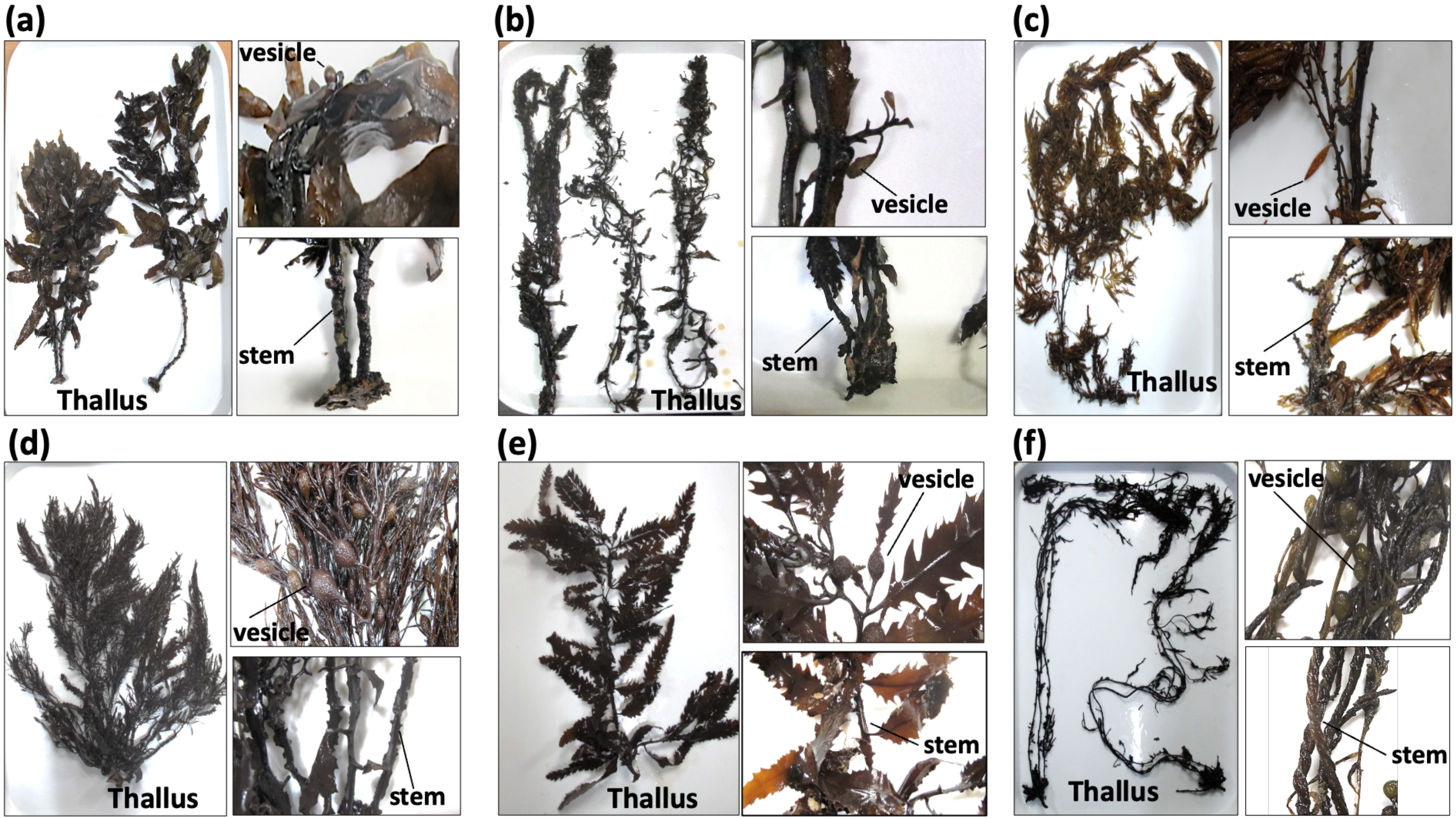

Figure S1. The morphological characteristics of collected samples. (a) *S. confusum*: spine-like appendages are occasionally found on the tip of elliptical shaped vesicle. The stems are cylindrical and spinose; (b) *S. fulvellum*: vesicles are spindle shaped and spine-like appendages are occasionally found. *S. fulvellum* has short cylindrical stem; (c) *S. horneri*: vesicles are spindle shaped. Stem has short thorns; (d) *S.*

macrocarpum: *S. macrocarpum* has spherical or obovate vesicles. The stems are cylindrical and spinose; (e) *S. serratifolium*: vesicles are spherical or obovate. The stems are cylindrical. The leaves are saw-teeth shaped; and (f) *S. siliquastrum*: *S. siliquastrum* has spherical or obovate vesicles. The stems are smooth and twisted.

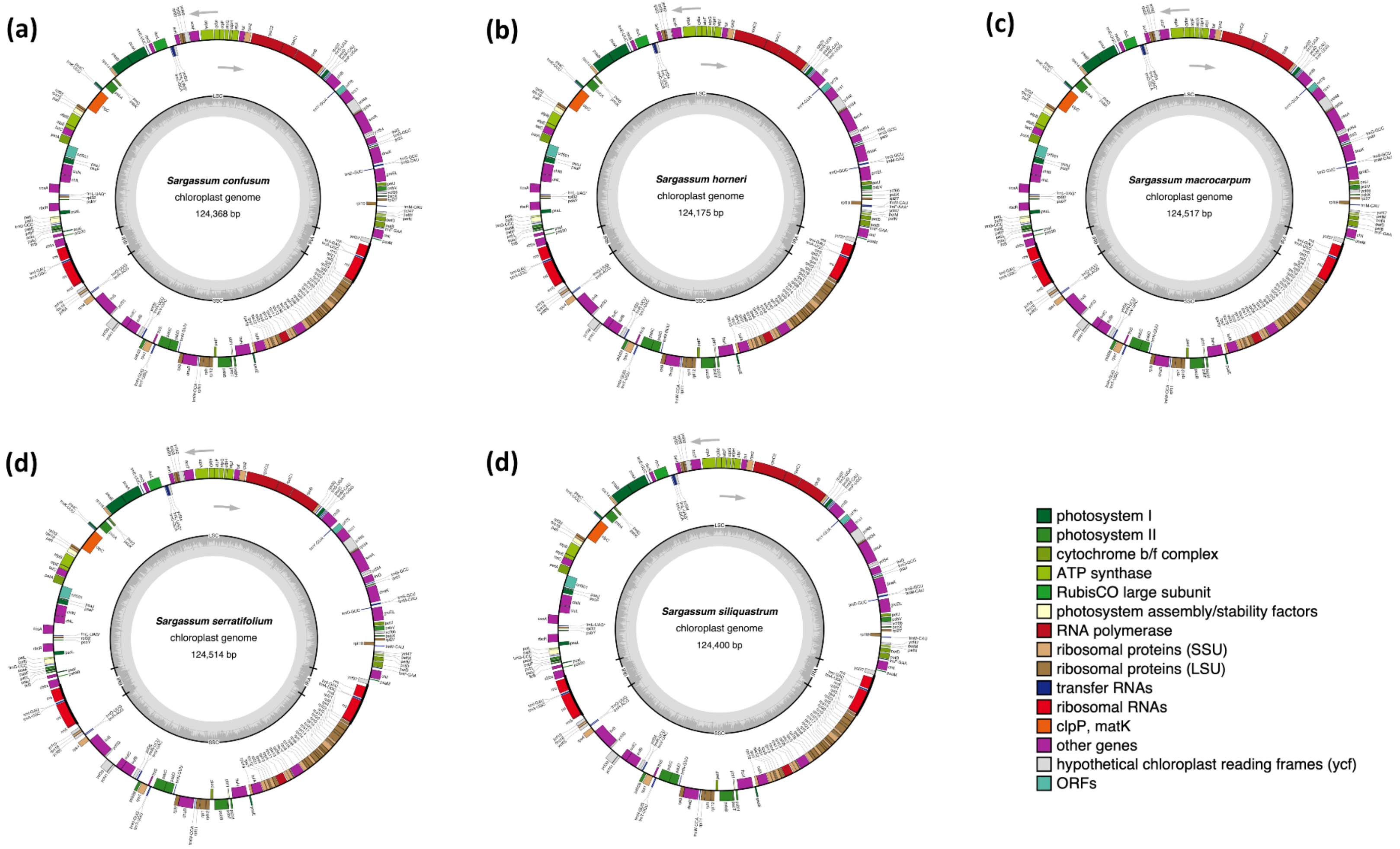

Figure S2. The chloroplast genome maps of (a) *S. confusum*, (b) *S. horneri*, (c) *S. macrocarpum*, (d) *S. serratifolium*, and (e) *S. siliquastrum*. The genes inside and outside of the circle are transcribed in the clockwise and counterclockwise directions, respectively. The grey circle on the inside shows the GC content. The colored boxes represent gene functional groups. The thick lines indicate the inverted repeats (IRa and IRb), which separate the genome into small (SSC) and large (LSC) single-copy regions.

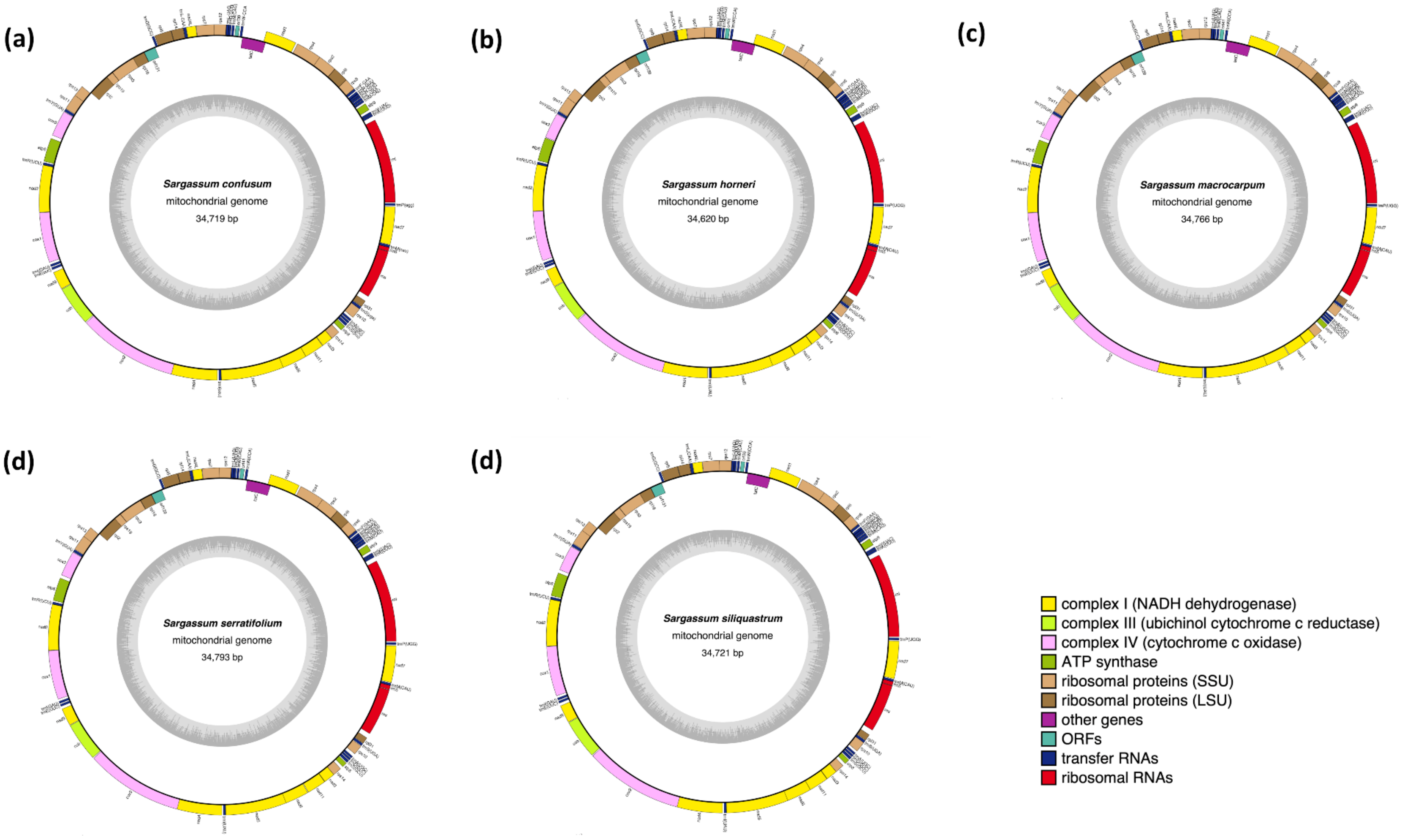

Figure S3. The mitochondrial genome maps of (a) *S. confusum*, (b) *S. horneri*, (c) *S. macrocarpum*, (d) *S. serratifolium*, and (e) *S. siliquastrum*. The genes inside and outside of the circle are transcribed in the clockwise and counterclockwise directions, respectively. The grey circle on the inside shows the GC content. The colored boxes represent gene functional groups.

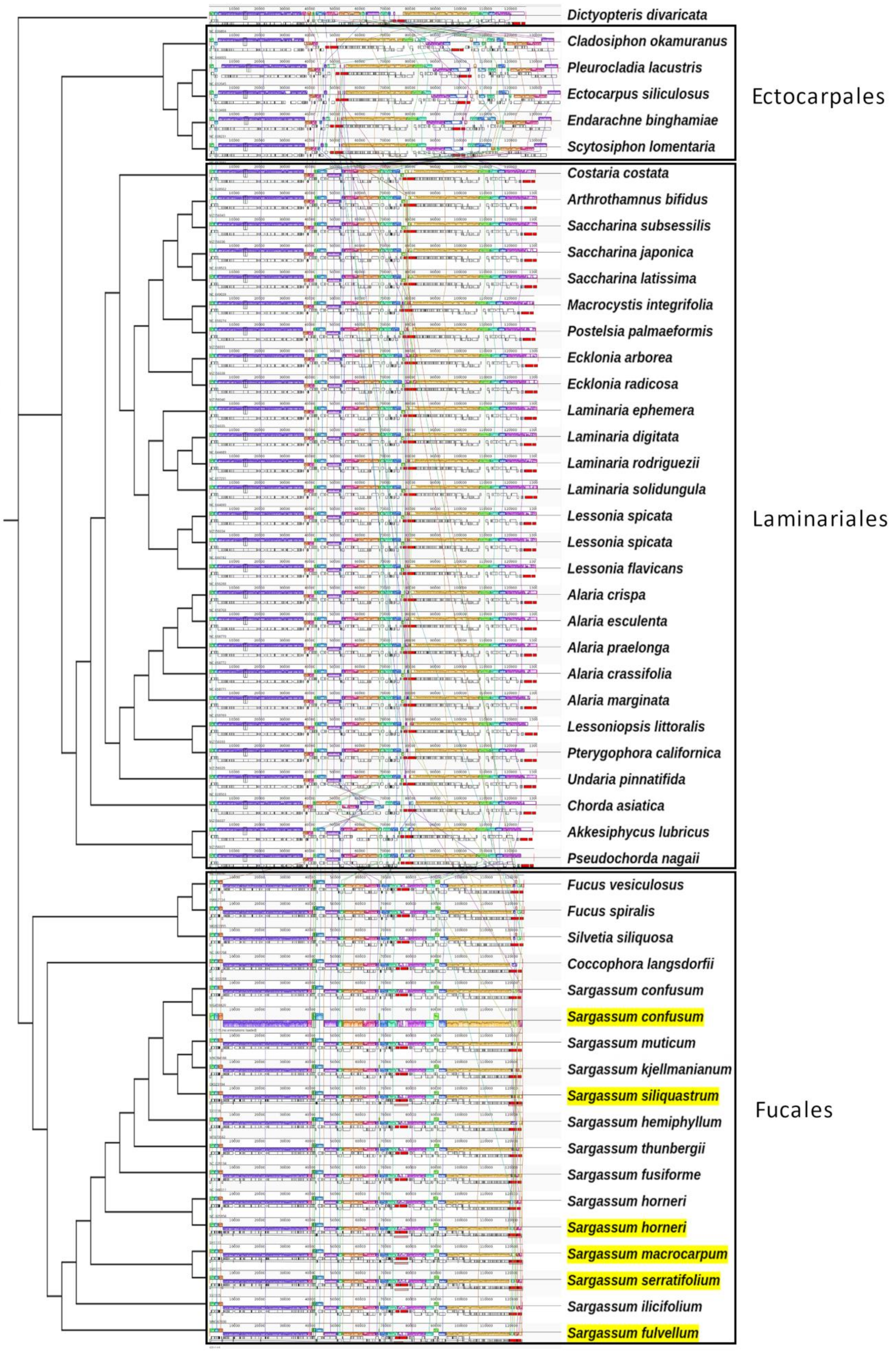

Figure S4. Gene rearrangements of Phaeophyceae 44 species chloroplast genomes. Local collinear blocks were colored to indicate syntenic regions.

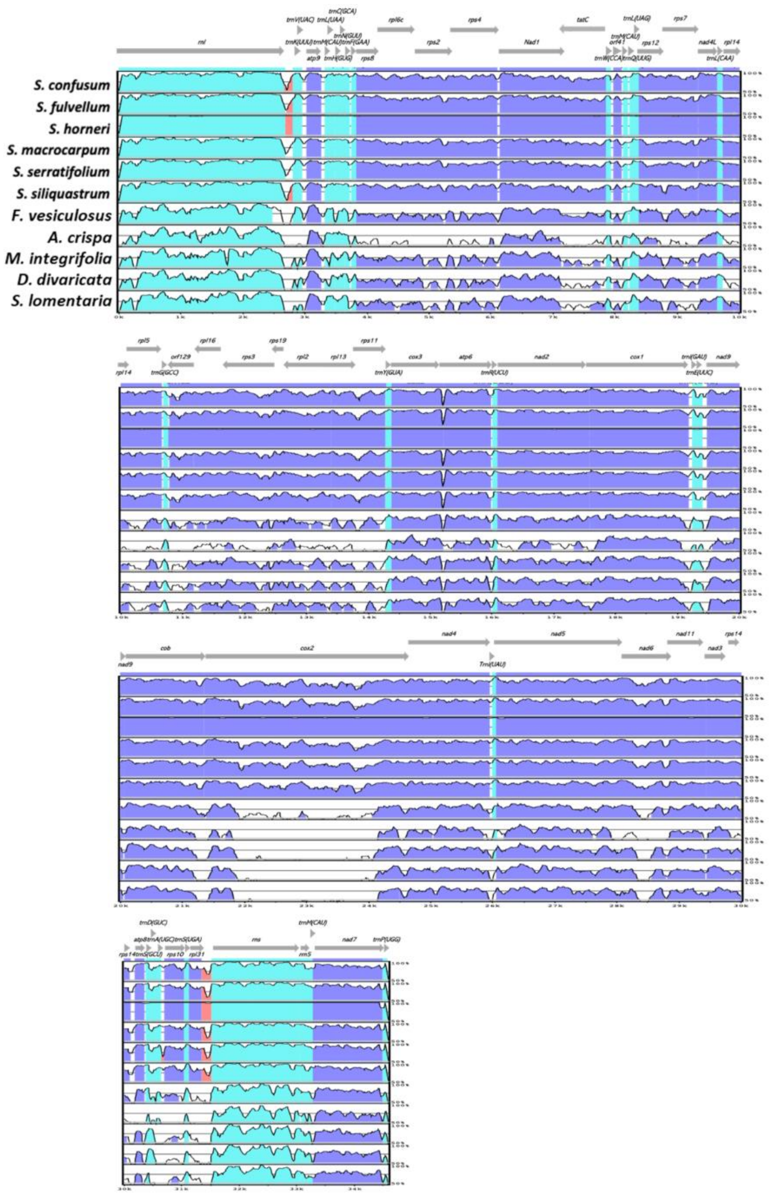

Figure S5. Alignment for sequence similarity among the 12 Phaeophyceae chloroplast genomes. Chloroplast genome of *S. horneri* (NC_024613) was used as the reference. Untranslated, conserved non-coding and coding regions were colored by skyblue, red, and blue, respectively.

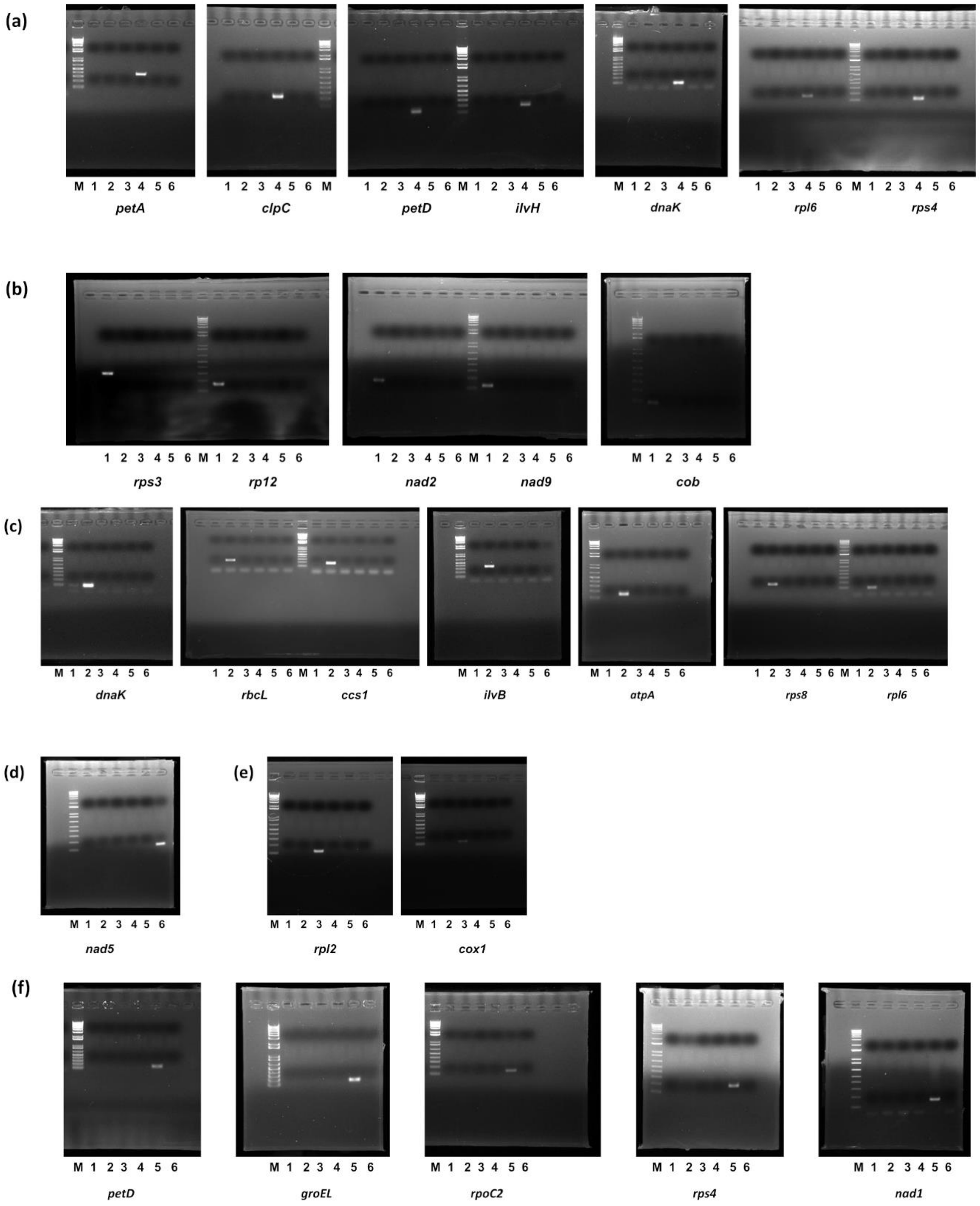

Figure S6. Full-length gels of Fig. 7. Target species are (a) *S. confusum*, (b) S. *fulvellum*, (c) *S. horneri*, (d) *S. macrocarpum*, (e) *S. serratifolium*, and (f) *S. siliquastrum*. Lane 1: S. *fulvellum*; 2: *S. horneri*; 3: *S. serratifolium*; 4: *S. confusum;* 5: *S. siliquastrum;* 6: *S. macrocarpum,* M: DNA ladder.
